# Building new knowledge: Celebrating the Wits School of Public Health (WSPH)

**DOI:** 10.3402/gha.v6i0.20241

**Published:** 2013-01-24

**Authors:** Laetitia C. Rispel, Sharon Fonn

**Affiliations:** Faculty of Health Sciences, University of the Witwatersrand, Johannesburg, South Africa

Notwithstanding worldwide aggregate improvements in the health of individuals and communities, (1, 2) overall progress has been marred by a multiplicity of factors. These include: the changing burden and complexity of disease profiles; the social, political, economic and environmental drivers of ill-health, unacceptable health inequalities between and within countries, and inadequate or poorly performing health systems (1, 3). Common shortcomings of contemporary health systems include fragmented, inappropriate, misdirected or poor quality care which mitigate against a comprehensive and balanced response to population health needs (1). All these challenges are more acute in sub-Saharan Africa, which faces additional problems of financial and human resource constraints and low investment in health research (3, 4).

In South Africa, health status indicators are poor relative to the country's economic development and health care expenditure (5). This is exacerbated by the sub-optimal performance of the health system, particularly at the district level (6–9). Significant efforts are required to overcome the HIV and TB epidemics, address persistent health inequalities, improve the quality of health care provision, and improve health outcomes (6). Schools of Public Health in Africa have a critical role to play in responding to these many systemic issues that confound improvements in population health. While solutions are complex, key priorities for action include: revitalising *primary health care* (PHC); addressing the *crisis in human resources for health*; conducting *relevant research* to respond to the advanced health and social transitions underway in the country, increase our understanding of barriers to improved population health, and to the performance of the health system; and *policy advocacy and engagement* that promotes effective stewardship and leadership of the health system (10–16).

In this supplement of *Global Health Action* we present a contribution of the School of Public Health at the University of the Witwatersrand (Wits) in Johannesburg to these critical issues. The mission of the Wits School of Public Health (WSPH) is to respond to the public health challenges facing South and sub-Saharan Africa, through research, the production of skilled, critical and adaptable graduates, and contributing to international scholarship and capacity building. The supplement brings together two commentaries and 23 scholarly articles that share historical developments at the WSPH, present methodological innovations in public health and explore themes that have captured global attention in recent years: improving the health of communities and of workers; understanding population health risk factors; human resources for health; and optimising health system performance.

## Approach and methods

In January 2012, the WSPH obtained funding from a strategic University fund for the supplement. The overall goal of the supplement was to bring together a series of papers exploring trends, developments and new directions for scientific enquiry regarding public health in South Africa and in the region, while speaking to an international public health audience. Another important goal was to address a critical human resource challenge in the region, that of nurturing and developing the next generation of African scholars.

In March 2012, we invited all staff (including honorary staff), post-graduate students, alumni and WSPH collaborators to submit abstracts that correspond to the diverse and multi-disciplinary areas of endeavour of the School. An intensive review process provided inputs and support on how to develop these abstracts further. Thereafter full papers were elicited and authors were invited to a writing workshop. In preparation for the workshop, pairs of authors were asked to peer review each other's papers. Simultaneously, senior academics provided external expert review. This provided the opportunity for junior academics to compare their reviews with that of senior academics. The two-day writing retreat, where mentors were available to work with authors, provided guidance for junior authors and a supportive environment and time away for all authors to focus on their writing. A further process of paper revision and refinement followed, prior to the authors submitting their papers to *Global Health Action*.

For each paper, we selected national and international peer reviewers to ensure that local content would be accurate and that papers would be sound, relevant, and interesting to an international audience as well. An extensive peer review process was followed, including re-assessment by the same reviewers in some instances. The papers were accepted for publication and went to production only after peer-reviewers recommended acceptance. The result is this supplement in which we are *building new knowledge* and which includes a number of first time, first-author publications from African academics and post graduate students.

## Themes and focus

The two commentaries by our predecessors John Gear (17) and William Pick (18), trace the historical developments of public health at Wits University. Both authors highlight: the importance of an enduring set of values of human rights, equity and social justice; the need to respond to the broader political and social context in the development of public health; the unique focus on population health rather than individual health; the multi-disciplinary nature of the discipline; and the methodological, educational and health service innovations that characterised the first two decades of community (or public) health at the University. As is still the case today, the WSPH prides itself on the diversity of its research and teaching endeavours, its involvement and influence on many aspects of public health ranging from shaping national and international health policy, through health service delivery to the development of human resources for health.

Many issue highlighted in these historical overviews are common strands running through the subsequent papers: the centrality of the measurement sciences to the discipline of public health; improving the health of communities and of workers; understanding population health risk factors; concerns with policy design and implementation; challenges related to human resources for health; and optimising health system performance.

### Measurement sciences and health determinants

Measurement sciences are fundamental to public health. The WSPH both has strong research and training capacity in this area. In the first paper in this theme, Chirwa et al. (19) investigate the degree to which misclassification in infectious disease modelling can affect research findings and describe how household composition dynamics are important in infectious diseases that have long incubation periods. In light of limited information on long-term fertility transitions of refugee populations, Williams et al. (20) use demographic methods to examine the changes in the fertility of former Mozambican self-settled refugees over a period of 17 years and compare their overall fertility patterns with those of an established community in rural South Africa. The authors found that refugees ‘take on’ the fertility patterns of the host communities, but point out that further declines in fertility will only occur by addressing endemic poverty in the area and increasing education and job opportunities for women.

Both Sartorius (21) and Musenge et al. (22) explore spatial analysis as a method for understanding determinants of mortality at household, village and district level in relation to all cause and HIV/TB mortality respectively. Both papers draw out the policy relevance of this approach and identify social determinants of health as important in predicting mortality. Community characteristics and in particular socio-economic status and childhood mortality in Tanzania are explored by Nattey et al. (23). The authors explore the usefulness of a mortality concentration index and comparing mortality outcomes by wealth quintiles. They demonstrate the association between under-five mortality on the one hand and household socio-economic inequalities and maternal education on the other hand. Ramsoomar et al. (24) examine the prevalence of lifetime alcohol use during adolescence in an urban township of Johannesburg and its association with child and maternal socio-demographic factors. The findings suggest that gender, maternal education, and socio-economic status are predictors of alcohol use during adolescence.

### Training for transformation

Two articles in this supplement address training. Christofides et al. (25) describe a new Master of Public Health (MPH) programme at the WSPH that focuses on communication for social change. The MPH programme acknowledges the need to train graduates in an evidence-based approach to develop and evaluate interventions to improve population health through policy changes, the environment in which healthy choices are made and individual behaviours. The authors argue for investments in institution building for African programmes in Africa for Africans. The article by Khan et al. (26) reviews the response of medical schools in one South African province to the need to transform the race and gender make up of students and graduates. They note the progress in redressing historical disparities and inequities in terms of race and gender, but point out that further efforts are needed to ensure that student intakes and graduations are in line with the South African population profile.

### Occupational health

Mining has a long history in South Africa and the WSPH has a strong focus on occupational health. Neat compartmentalisation into categories however does not describe reality. The article by Ndlovu et al. (27) illustrates that commercial exploitation of mineral resources has health consequences for the surrounding communities. However, these communities are further disadvantaged by insufficient compensation and measures that address the source of the problem. Nelson (28) explores the exposure to silica in various forms of mining in South Africa and its health consequences. The author confirms the hazards of silica exposure and the inadequate responses from the employers, the possible risk of exposure to asbestos in both platinum and diamond miners, exacerbated by the inadequacy of exposure information. Banyini et al., (29) explore why miners who could benefit from post mortem autopsy and related compensation under-utilize this potential benefit, using in-depth qualitative methods. The authors identify a range of socio-cultural barriers to obtain consent for an autopsy, but note that multifaceted awareness and knowledge-building interventions are possible and should be implemented with healthy mine-workers.

### Optimal performance of the health system

In light of the international discourse on universal health care coverage, Govender et al. (30) assess the South African government employee health insurance scheme and describe the factors associated with low uptake of the scheme. The authors note that the barriers to enrolment include insufficient information, unaffordability of payments and perceived administrative complexity. While the conditions around the existing scheme are not directly comparable with plans in South Africa to set up a national health insurance (NHI) system, the article illustrates the paradox of low-uptake of health insurance among population groups with the greatest potential health need, thus informing planning around the proposed NHI.

Rural populations are often confronted with poor access to services and du Toit et al. (31) explore the degree to which there is consensus about measures needed to improve rural health service provision. Although not unique to rural areas, their findings identify human resources for health as an important constraint. Blaauw et al., (32) describe health worker job satisfaction in a multi-country study. Of interest in this study, and as yet insufficiently explained, is the finding that notwithstanding better working conditions in South Africa compared to Tanzania and Malawi, South African health care providers are the least satisfied in their jobs. Ditlopo et al. (33) explore a financial incentive that was introduced to improve nurses’ salaries in the South African public health sector. They illustrate the unintended negative consequences that can occur when policy development is not well aligned with the health sector's capacity to implement such policies. The article by Doherty et al. (34) describes the careful planning and consultation with multiple stakeholders around the introduction of a new cadre of health care provider, the clinical associate, in South Africa to avoid conflict among different cadres. The clinical associates are meant to improve capacity and care at district hospital level. The article concludes that while initially successful the way forward for this category of health worker is not assured.

Another challenge facing the South African health sector is how systems can be strengthened in the face of vertical programme implementation. Kawonga et al. (35) explore, through the lens of health information systems, how staff tasked with overall district health system responsibilities interact with those responsible for HIV services. The authors conclude that reporting systems of vertical services may undermine the overall goal of health systems strengthening.

Providing ongoing care for chronic conditions is becoming increasingly important for South Africa, and indeed for other low and middle-income countries, particularly those in sub-Saharan Africa. Sengayi et al. (36) explore predictors of loss to follow up in children on HIV treatment and found a curious relationship between loss to follow-up and being cared for by their mothers. They raise the question of differential access to treatment between children and their mothers. Chronic care is also an important service for the ageing population in South Africa. Gómez-Olivé et al.'s (37) survey of people over 50 years of age living in rural areas suggest high levels of chronic health conditions associated with higher levels of health care use. The authors caution that current health services in South Africa are inadequately prepared for the management of chronic conditions.

Research by Kimani-Murage (38) in the same rural area looked at possible predictors of chronic ill-health outcomes associated with metabolic syndrome by investigating the nutritional status of children. A combination of early stunting and adolescent obesity found in this area may predict an important chronic disease epidemic in the future. Bertram et al. (39) also predict an increase in the burden of chronic ill-health by using existing data to estimate the Years Lost due to Disability associated with diabetes. Providing health care services for patients which chronic health problems is a challenge and Ndou et al. (40) assess the role of community health workers in providing this care. They conclude, not surprisingly, that as for any intervention with community health workers, training, supervision, and operational support are required. Nxumalo et al. (41) explore various models of community health worker programmes, and find that coherent and stable communities, compared with communities of great heterogeneity and characterized by high levels of migration, are easier to work in. The authors note that support for community health workers is important.

## Conclusion

South Africa, as is the case in other African countries, faces formidable public health challenges, some of which have been explored in this supplement. At the same time, the supplement illustrates the importance of building a vibrant African academy, able to lead high quality, multidisciplinary research that generates new knowledge, is policy-relevant and that makes a positive impact on public and population health. The articles are authored by individuals from different disciplinary backgrounds and with a range of theoretical approaches, ranging from the social to the bio-medical sciences, competent in qualitative and/or quantitative research methods.

As is the case with our parent university, the WSPH is cognisant of the rapid and ongoing changes in the global, regional and national context and the need to respond to the profound public health challenges of the twenty-first century. Hence, the WSPH combines skills and approaches that respond to the advanced health and social transitions underway, emphasising the need to understand health policy and health systems and the need for advanced skills to develop, test, cost and evaluate interventions for broader population health improvements in South Africa and in the whole of Africa. We take pride in our contribution to the expansion of public health knowledge since democracy in South Africa, and in the growth and development of Public Health at Wits.

The supplement also underscores the need for greater investment in African Schools of Public health. First, through their training programmes, these Schools play a critical role in addressing critical human resource needs in the region and in building the next generation of practitioners and public health leaders. Second, investment will enable these Schools to enhance their capacity to lead globally-competitive research. Such research will ensure the production of new knowledge and guidance on how best to: address many of the causes of premature mortality or morbidity among Africans; implement proven interventions to prevent premature morbidity and mortality; and design and implement the reforms necessary for a well-functioning health system. Further research is also needed to determine why leadership and management action to implement known and effective public health interventions is found wanting at almost all levels of the health system.

In conclusion, the supplement highlights the need for strong government stewardship and leadership, in promoting the health of people, through action on the social determinants of health and action to improve the performance of the health system. A critical imperative is to use our collective knowledge to improve population health of those most in need, through collaboration and effective partnerships, regionally, nationally and internationally, across various sectors and across various stakeholders.

*Laetitia C. Rispel* Current head of the Wits, School of Public Health*Sharon Fonn* Past head of the Wits, School of Public Health, 2003–2011 Faculty of Health Sciences University of the Witwatersrand, Johannesburg South Africa
